# Supplementary material for: Metformin Protects Rat Skeletal Muscle from Physical Exercise-Induced Injury
Source: Biomedicines. 2023 Aug 22;11(9):2334. doi: 10.3390/biomedicines11092334 (PMC10525561; doi:10.3390/biomedicines11092334)
Supplement: Supplementary file 1 [file biomedicines-11-02334-s001.zip › Figure S5.pdf]

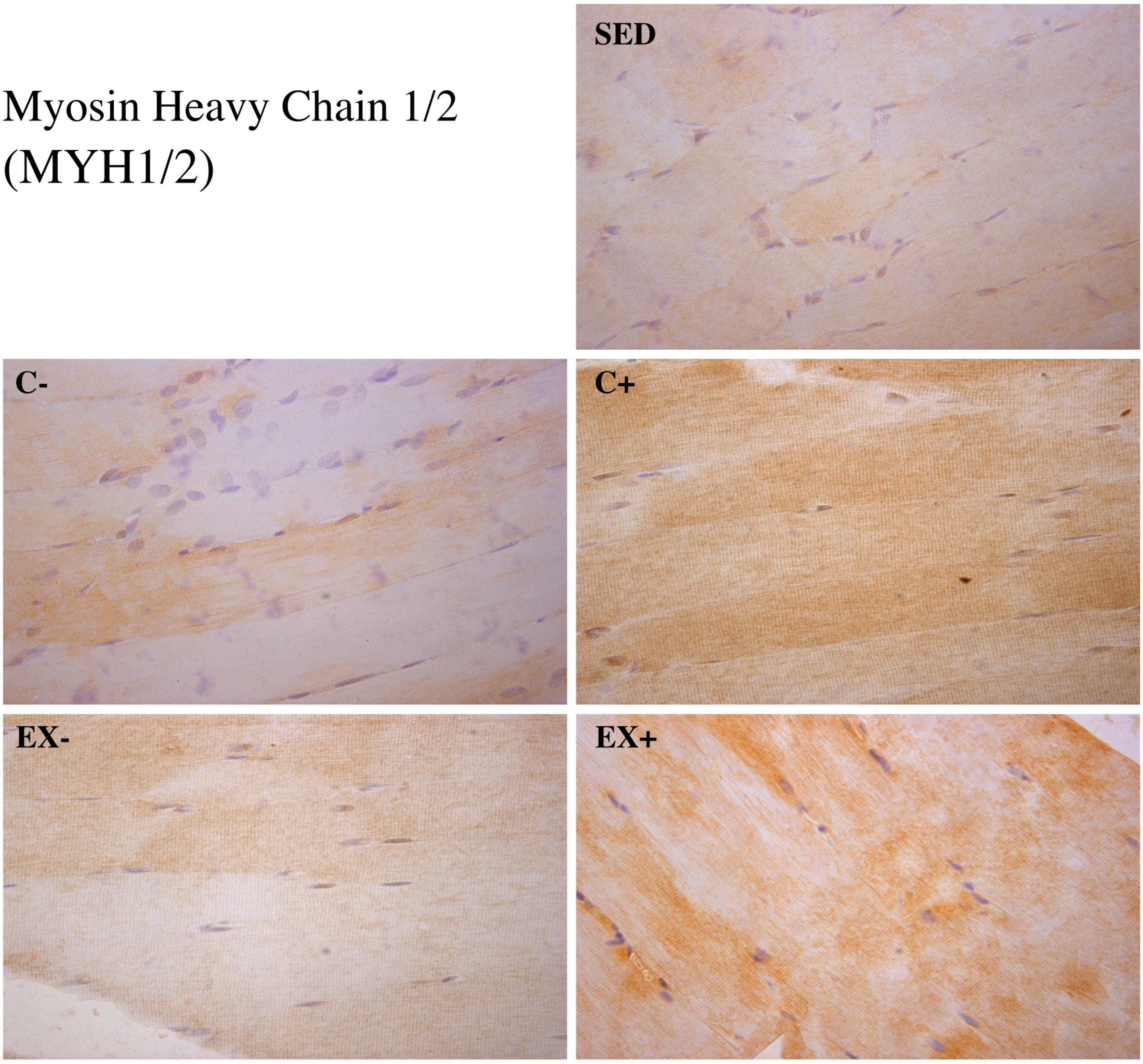

Figure S5. Representative immunohistochemical staining of Myosin Heavy Chain 1/2 (MYH1/2) from muscle tissue of experimental groups. Magnification 20X.
