# Supplementary material for: Metformin Protects Rat Skeletal Muscle from Physical Exercise-Induced Injury
Source: Biomedicines. 2023 Aug 22;11(9):2334. doi: 10.3390/biomedicines11092334 (PMC10525561; doi:10.3390/biomedicines11092334)

Identity of the experimental samples: total muscle lysates
Image captured by using ChemiDoc™ Touch Image System (Bio-Rad)
Figure generated from this original image: Fig 7a/b (AMPK) - replicate 1

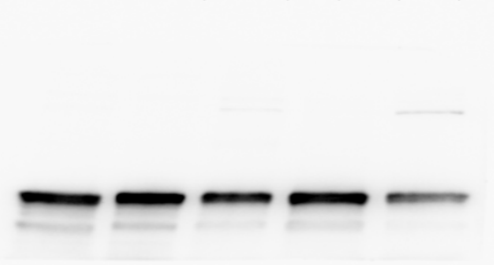

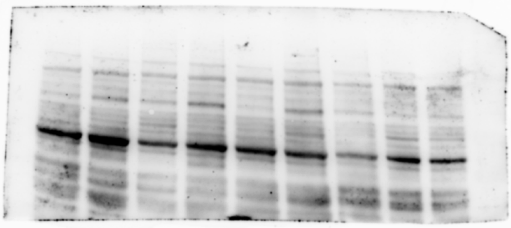

Identity of the experimental samples: total muscle lysates
Image captured by using ChemiDoc™ Touch Image System (Bio-Rad)
Figure generated from this original image: Fig 7a/b (AMPK) - replicate 2

Identity of the experimental samples: total muscle lysates
Image captured by using ChemiDoc™ Touch Image System (Bio-Rad)
Figure generated from this original image: Fig 7a/b (AMPK) - replicate 3

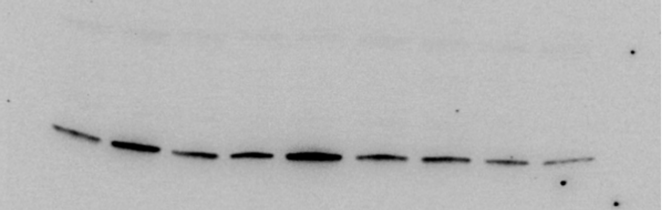

Identity of the experimental samples: total muscle lysates
Image captured by using ChemiDoc™ Touch Image System (Bio-Rad)
Figure generated from this original image: Fig 7a/b (p-AMPK) - replicate 1

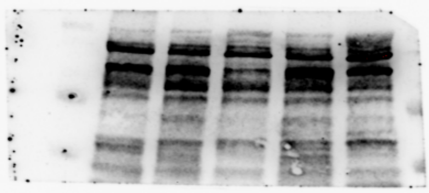

Identity of the experimental samples: total muscle lysates
Image captured by using ChemiDoc™ Touch Image System (Bio-Rad)
Figure generated from this original image: Fig 7a/b (p-AMPK) - replicate 2

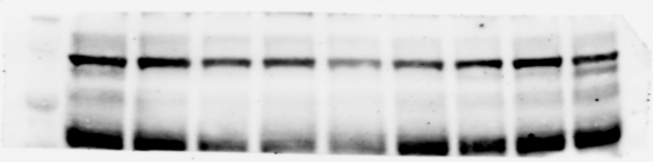

Identity of the experimental samples: total muscle lysates
Image captured by using ChemiDoc™ Touch Image System (Bio-Rad)
Figure generated from this original image: Fig 7a/b (p-AMPK) - replicate 3

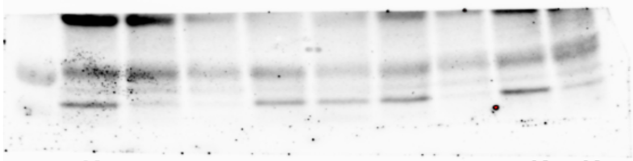

Identity of the experimental samples: total muscle lysates
Image captured by using ChemiDoc™ Touch Image System (Bio-Rad)
Figure generated from this original image: Fig 8 (ACCß) - replicate 1

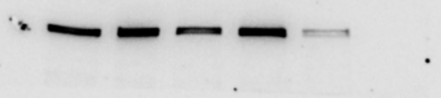

Identity of the experimental samples: total muscle lysates
Image captured by using ChemiDoc™ Touch Image System (Bio-Rad)
Figure generated from this original image: Fig 8 (ACCß) - replicate 2

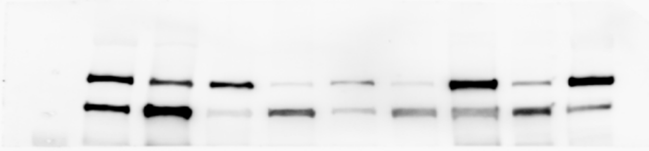

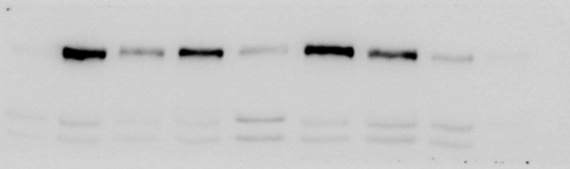

Identity of the experimental samples: total muscle lysates
Image captured by using ChemiDoc™ Touch Image System (Bio-Rad)
Figure generated from this original image: Fig 8 (ACCß) - replicate 3

Identity of the experimental samples: total muscle lysates
Image captured by using ChemiDoc™ Touch Image System (Bio-Rad)
Figure generated from this original image: Fig 8 (p-ACC) - replicate 1

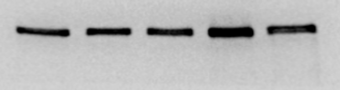

Identity of the experimental samples: total muscle lysates
Image captured by using ChemiDoc™ Touch Image System (Bio-Rad)
Figure generated from this original image: Fig 8 (p-ACC) - replicate 2

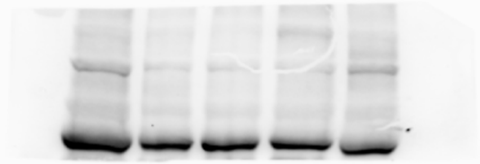

Identity of the experimental samples: total muscle lysates
Image captured by using ChemiDoc™ Touch Image System (Bio-Rad)
Figure generated from this original image: Fig 8 (p-ACC) - replicate 3

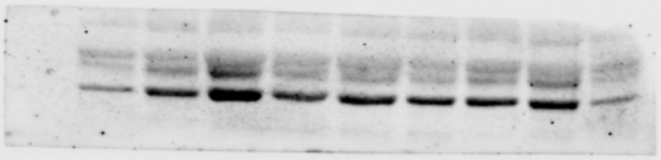

Identity of the experimental samples: muscle mitochondrial fraction.
Image captured by using ChemiDoc™ Touch Image System (Bio-Rad)
Figure generated from this original image: Fig 9a - replicate 1

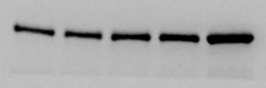

Identity of the experimental samples: muscle mitochondrial fraction.
Image captured by using ChemiDoc™ Touch Image System (Bio-Rad)
Figure generated from this original image: Fig 9a - replicate 2

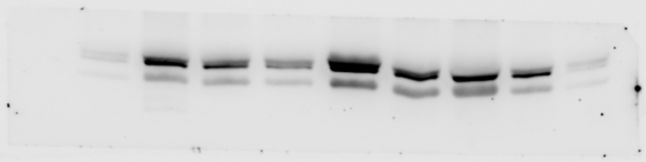

Identity of the experimental samples: muscle mitochondrial fraction.
Image captured by using ChemiDoc™ Touch Image System (Bio-Rad)
Figure generated from this original image: Fig 9a - replicate 3

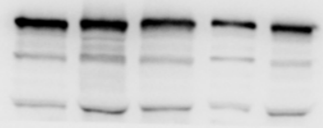

Identity of the experimental samples: muscle mitochondrial fraction.
Image captured by using ChemiDoc™ Touch Image System (Bio-Rad)
Figure generated from this original image: Fig 9b - replicate 1

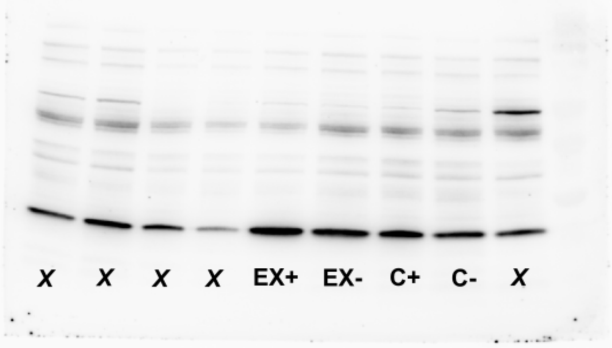

Identity of the experimental samples: muscle mitochondrial fraction.
Image captured by using ChemiDoc™ Touch Image System (Bio-Rad)
Figure generated from this original image: Fig 9b - replicate 2

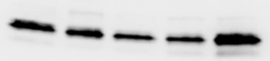

Identity of the experimental samples: muscle mitochondrial fraction.
Image captured by using ChemiDoc™ Touch Image System (Bio-Rad)
Figure generated from this original image: Fig 9b - replicate 3

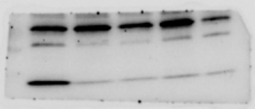

Identity of the experimental samples: total muscle lysates
Image captured by using ChemiDoc™ Touch Image System (Bio-Rad)
Figure generated from this original image: Fig 10a (p-AKT) - replicate 1

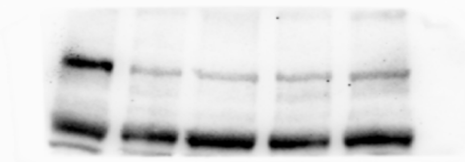

Identity of the experimental samples: total muscle lysates
Image captured by using ChemiDoc™ Touch Image System (Bio-Rad)
Figure generated from this original image: Fig 10a (p-AKT) - replicate 2

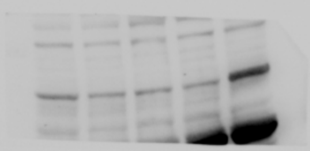

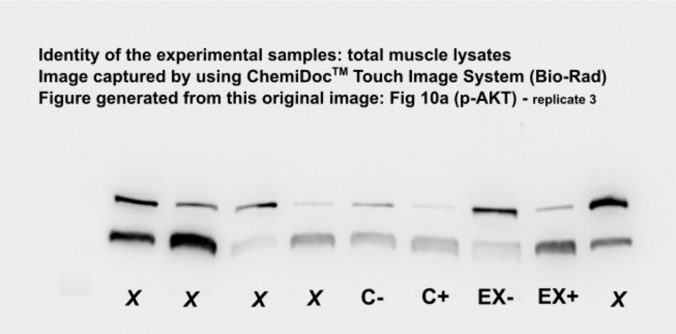
Identity of the experimental samples: total muscle lysates
Image captured by using ChemiDoc^{TM} Touch Image System (Bio-Rad)
Figure generated from this original image: Fig 10a (p-AKT) - replicate 3
X X X X C- C+ EX- EX+ X

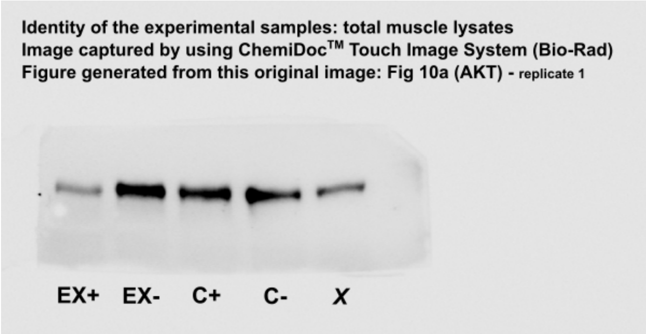
Identity of the experimental samples: total muscle lysates
Image captured by using ChemiDoc^{TM} Touch Image System (Bio-Rad)
Figure generated from this original image: Fig 10a (AKT) - replicate 1
EX+ EX- C+ C- X

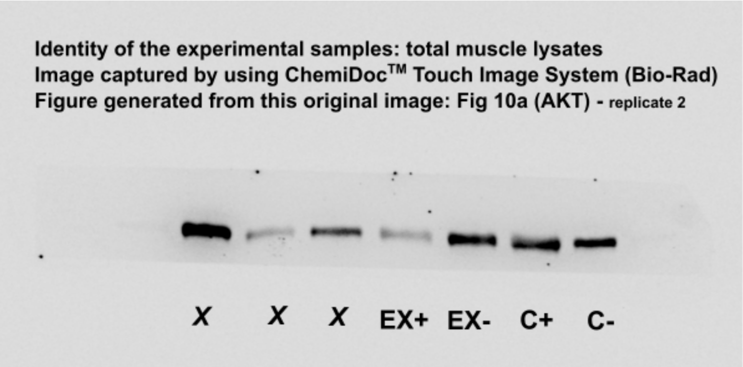
Identity of the experimental samples: total muscle lysates
Image captured by using ChemiDoc^{TM} Touch Image System (Bio-Rad)
Figure generated from this original image: Fig 10a (AKT) - replicate 2
X X X EX+ EX- C+ C-

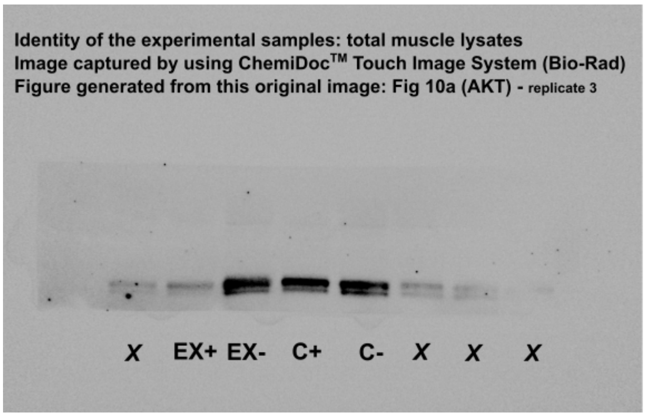
Identity of the experimental samples: total muscle lysates
Image captured by using ChemiDoc^{TM} Touch Image System (Bio-Rad)
Figure generated from this original image: Fig 10a (AKT) - replicate 3
X EX+ EX- C+ C- X X X

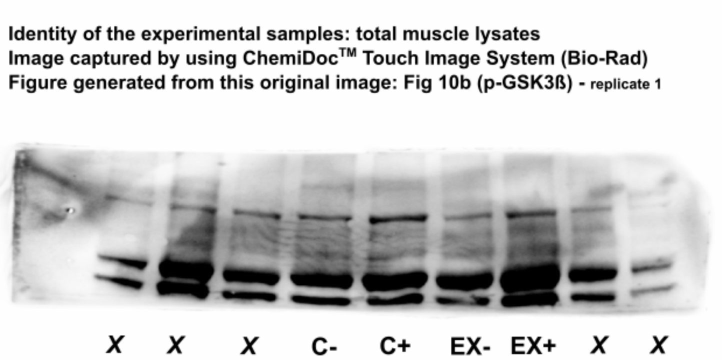
Identity of the experimental samples: total muscle lysates
Image captured by using ChemiDoc^{TM} Touch Image System (Bio-Rad)
Figure generated from this original image: Fig 10b (p-GSK3ß) - replicate 1
X X X C- C+ EX- EX+ X X

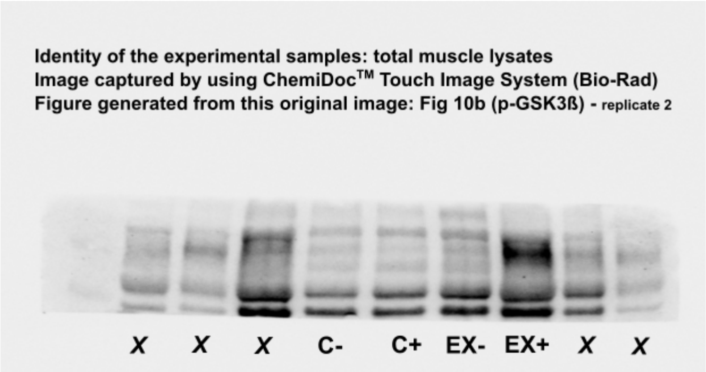
Identity of the experimental samples: total muscle lysates
Image captured by using ChemiDoc^{TM} Touch Image System (Bio-Rad)
Figure generated from this original image: Fig 10b (p-GSK3ß) - replicate 2
X X X C- C+ EX- EX+ X X

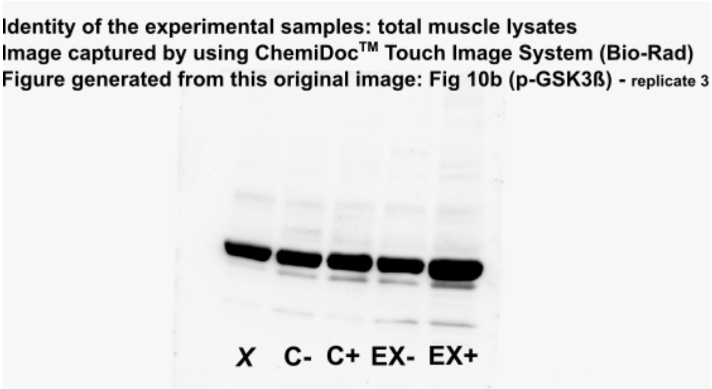
Identity of the experimental samples: total muscle lysates
Image captured by using ChemiDoc^{TM} Touch Image System (Bio-Rad)
Figure generated from this original image: Fig 10b (p-GSK3ß) - replicate 3
X C- C+ EX- EX+

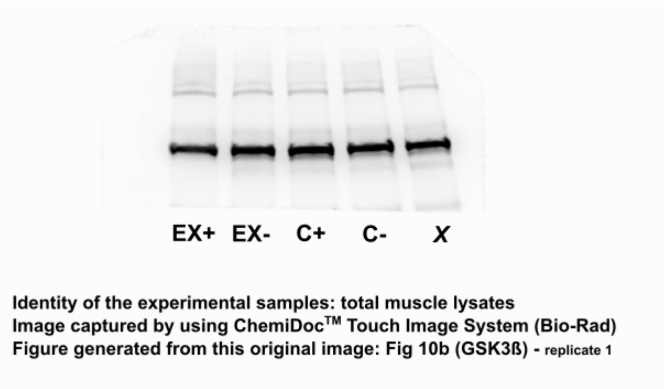
EX+ EX- C+ C- X
Identity of the experimental samples: total muscle lysates
Image captured by using ChemiDoc^{TM} Touch Image System (Bio-Rad)
Figure generated from this original image: Fig 10b (GSK3ß) - replicate 1

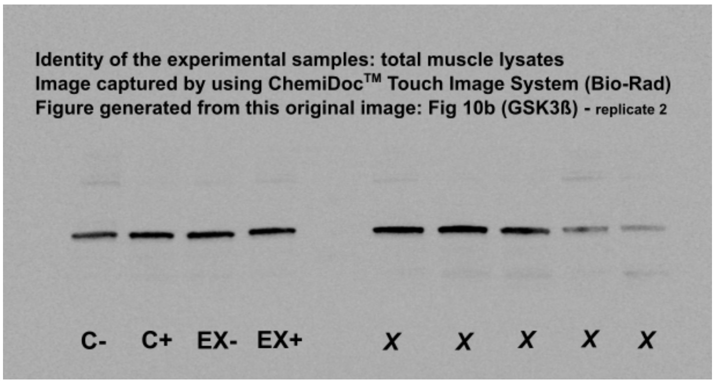
Identity of the experimental samples: total muscle lysates
Image captured by using ChemiDoc^{TM} Touch Image System (Bio-Rad)
Figure generated from this original image: Fig 10b (GSK3ß) - replicate 2
C- C+ EX- EX+ X X X X X

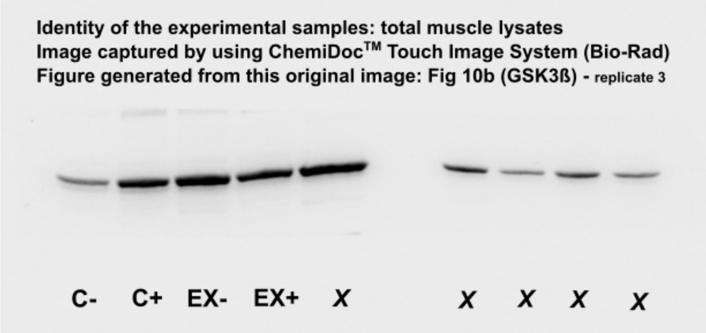
Identity of the experimental samples: total muscle lysates
Image captured by using ChemiDoc^{TM} Touch Image System (Bio-Rad)
Figure generated from this original image: Fig 10b (GSK3ß) - replicate 3
C- C+ EX- EX+ X X X X X

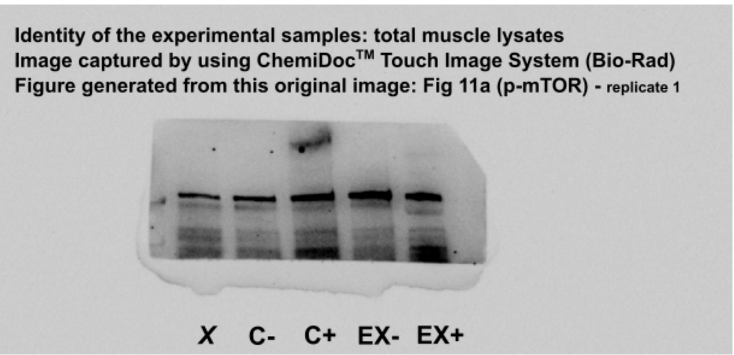
Identity of the experimental samples: total muscle lysates
Image captured by using ChemiDocTM Touch Image System (Bio-Rad)
Figure generated from this original image: Fig 11a (p-mTOR) - replicate 1
X C- C+ EX- EX+

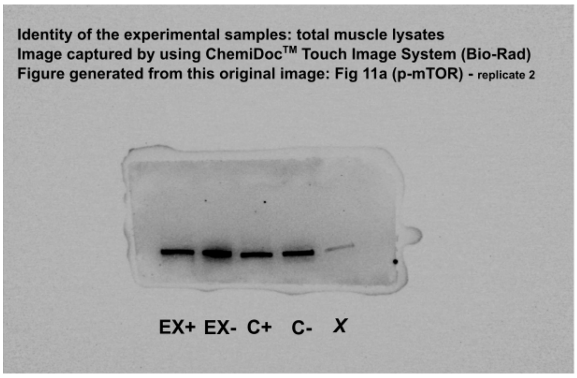
Identity of the experimental samples: total muscle lysates
Image captured by using ChemiDocTM Touch Image System (Bio-Rad)
Figure generated from this original image: Fig 11a (p-mTOR) - replicate 2
EX+ EX- C+ C- X

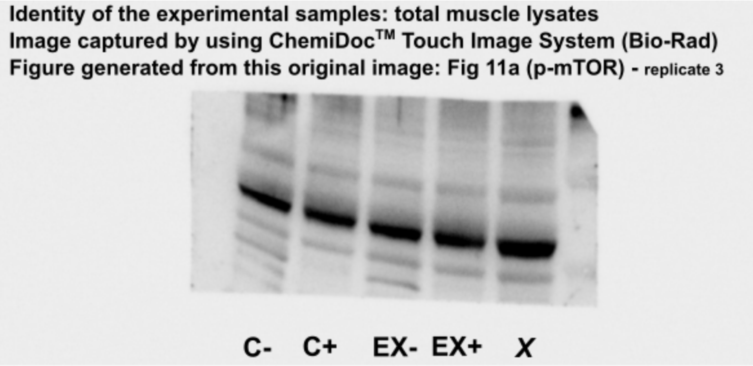
Identity of the experimental samples: total muscle lysates
Image captured by using ChemiDocTM Touch Image System (Bio-Rad)
Figure generated from this original image: Fig 11a (p-mTOR) - replicate 3
C- C+ EX- EX+ X

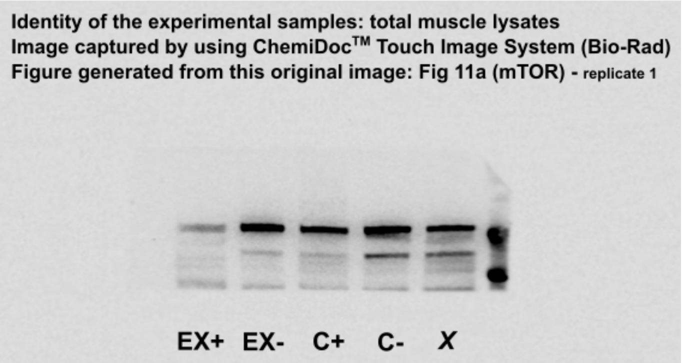
Identity of the experimental samples: total muscle lysates
Image captured by using ChemiDocTM Touch Image System (Bio-Rad)
Figure generated from this original image: Fig 11a (mTOR) - replicate 1
EX+ EX- C+ C- X

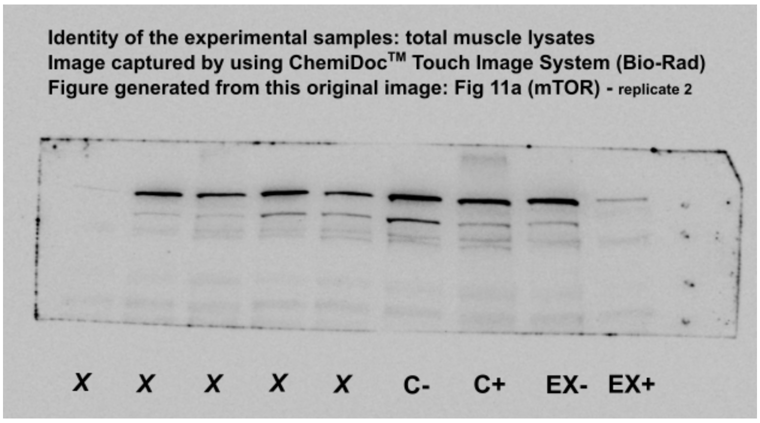
Identity of the experimental samples: total muscle lysates
Image captured by using ChemiDocTM Touch Image System (Bio-Rad)
Figure generated from this original image: Fig 11a (mTOR) - replicate 2
X X X X X C- C+ EX- EX+

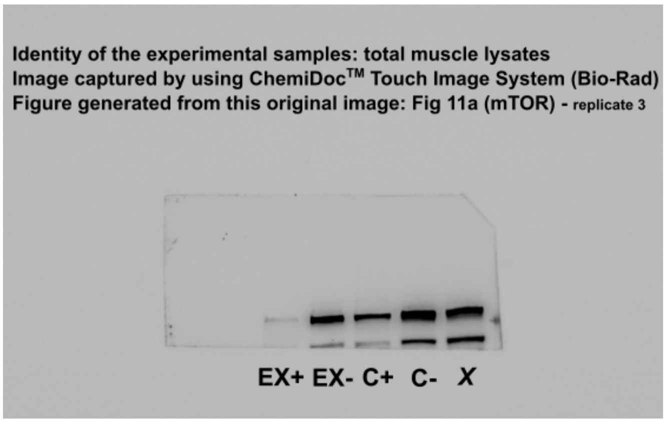
Identity of the experimental samples: total muscle lysates
Image captured by using ChemiDocTM Touch Image System (Bio-Rad)
Figure generated from this original image: Fig 11a (mTOR) - replicate 3
EX+ EX- C+ C- X

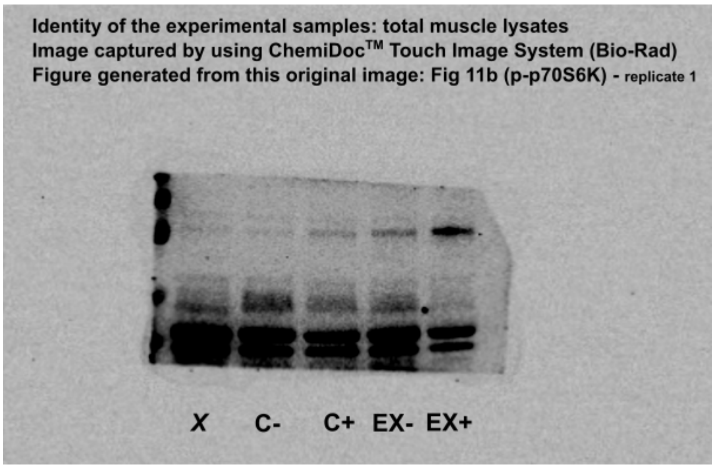
Identity of the experimental samples: total muscle lysates
Image captured by using ChemiDocTM Touch Image System (Bio-Rad)
Figure generated from this original image: Fig 11b (p-p70S6K) - replicate 1
X C- C+ EX- EX+

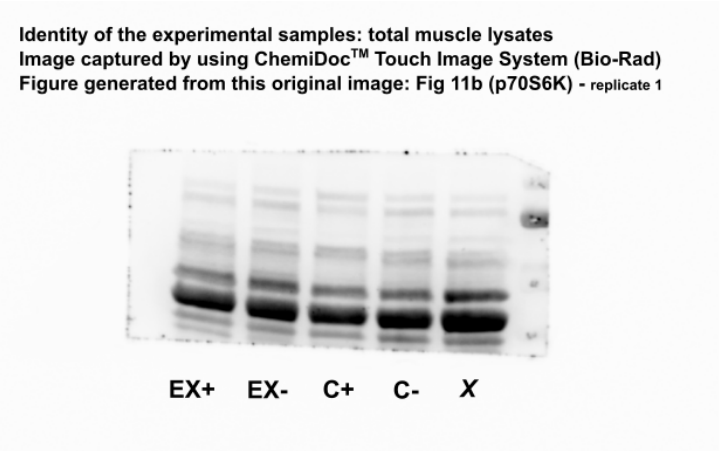
Identity of the experimental samples: total muscle lysates
Image captured by using ChemiDocTM Touch Image System (Bio-Rad)
Figure generated from this original image: Fig 11b (p70S6K) - replicate 1
EX+ EX- C+ C- X

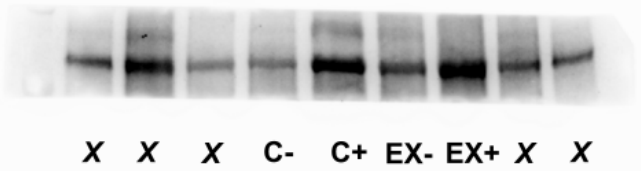

Identity of the experimental samples: total muscle lysates
Image captured by using ChemiDoc™ Touch Image System (Bio-Rad)
Figure generated from this original image: Fig 12 - replicate 1

Identity of the experimental samples: total muscle lysates
Image captured by using ChemiDoc™ Touch Image System (Bio-Rad)
Figure generated from this original image: Fig 12 - replicate 2

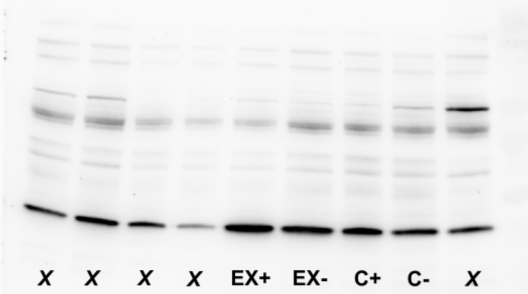

Identity of the experimental samples: total muscle lysates
Image captured by using ChemiDoc™ Touch Image System (Bio-Rad)
Figure generated from this original image: Fig 12 - replicate 3

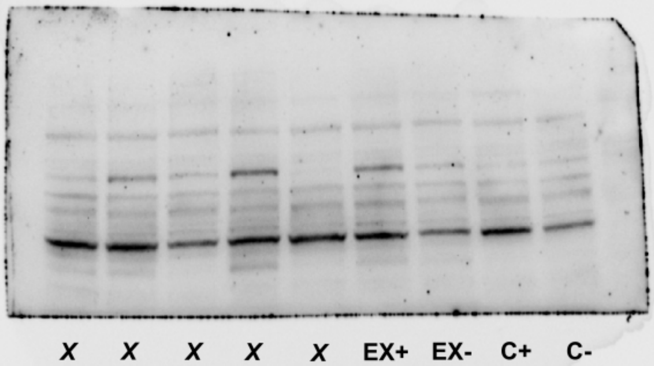

Identity of the experimental samples: total muscle lysates
Image captured by using ChemiDoc™ Touch Image System (Bio-Rad)
Figure generated from this original image: Fig 13a/b - replicate 1

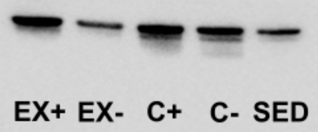

Identity of the experimental samples: total muscle lysates
Image captured by using ChemiDoc™ Touch Image System (Bio-Rad)
Figure generated from this original image: Fig 13a - replicate 2

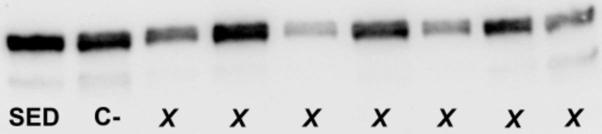

Identity of the experimental samples: total muscle lysates
Image captured by using ChemiDoc™ Touch Image System (Bio-Rad)
Figure generated from this original image: Fig 13a - replicate 2

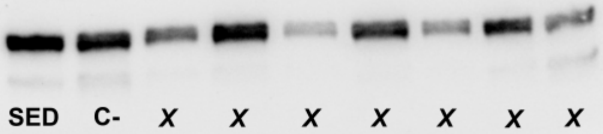

Identity of the experimental samples: total muscle lysates
Image captured by using ChemiDocTM Touch Image System (Bio-Rad)
Figure generated from this original image: Fig 13a - replicate 3

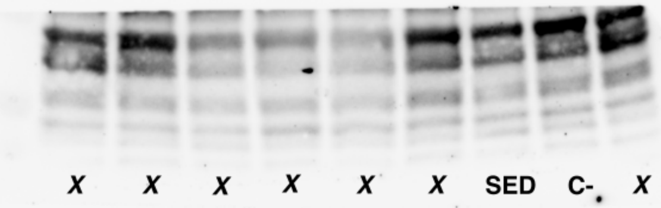

Identity of the experimental samples: total muscle lysates
Image captured by using ChemiDoc™ Touch Image System (Bio-Rad)
Figure generated from this original image: Fig 13b - replicate 2

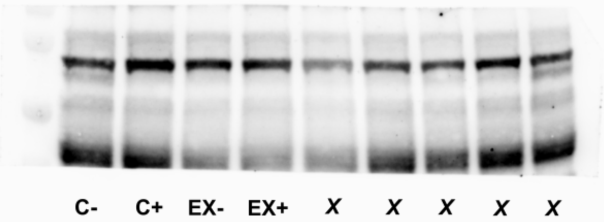

Identity of the experimental samples: total muscle lysates
Image captured by using ChemiDocTM Touch Image System (Bio-Rad)
Figure generated from this original image: Fig 13b - replicate 3

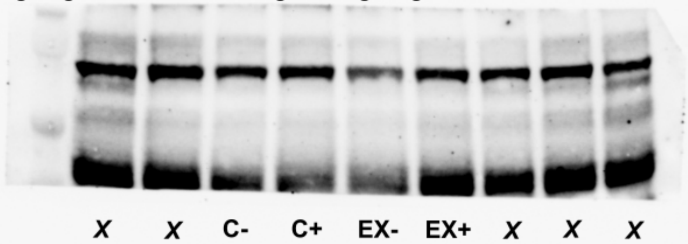

Identity of the experimental samples: total muscle lysates
Image captured by using ChemiDoc™ Touch Image System (Bio-Rad)
Figure generated from this original image: Fig 14a/b - replicate 1

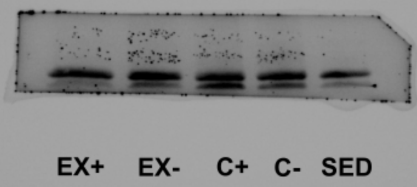

Identity of the experimental samples: total muscle lysates
Image captured by using ChemiDoc™ Touch Image System (Bio-Rad)
Figure generated from this original image: Fig 14a - replicate 2

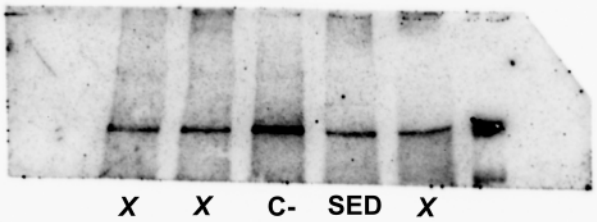

Identity of the experimental samples: total muscle lysates
Image captured by using ChemiDoc™ Touch Image System (Bio-Rad)
Figure generated from this original image: Fig 14a - replicate 3

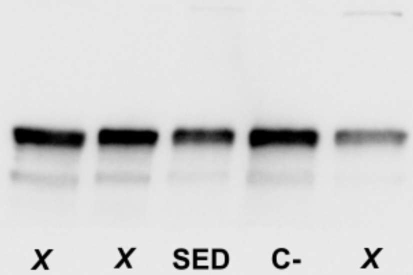

Identity of the experimental samples: total muscle lysates
Image captured by using ChemiDocTM Touch Image System (Bio-Rad)
Figure generated from this original image: Fig 14b - replicate 2

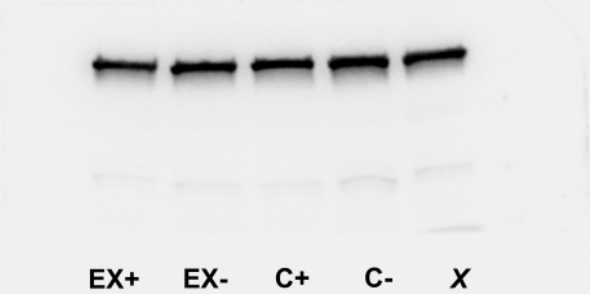

Identity of the experimental samples: total muscle lysates
Image captured by using ChemiDoc™ Touch Image System (Bio-Rad)
Figure generated from this original image: Fig 14b - replicate 3

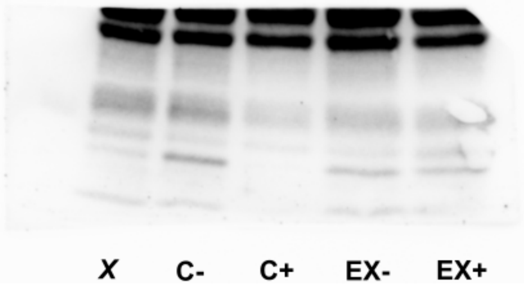

Identity of the experimental samples: total muscle lysates
Image captured by using ChemiDoc™ Touch Image System (Bio-Rad)
Figure generated from this original image: Fig 15a/b - replicate 1

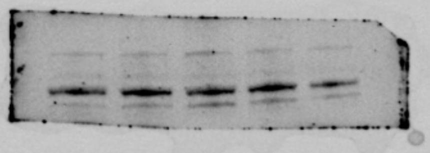

Identity of the experimental samples: total muscle lysates
Image captured by using ChemiDoc™ Touch Image System (Bio-Rad)
Figure generated from this original image: Fig 15a - replicate 2

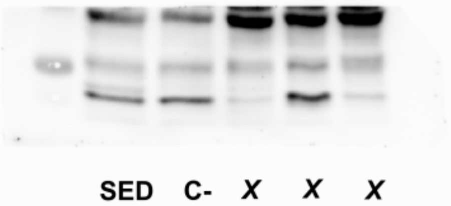

Identity of the experimental samples: total muscle lysates
Image captured by using ChemiDoc™ Touch Image System (Bio-Rad)
Figure generated from this original image: Fig 15a - replicate 3

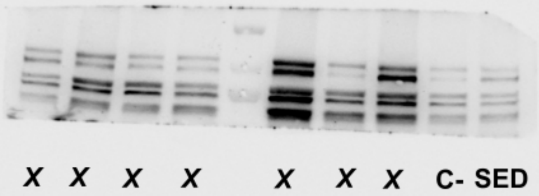

Identity of the experimental samples: total muscle lysates
Image captured by using ChemiDoc™ Touch Image System (Bio-Rad)
Figure generated from this original image: Fig 15b - replicate 2

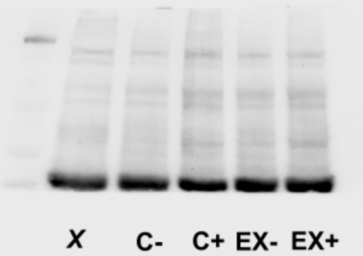

Identity of the experimental samples: total muscle lysates
Image captured by using ChemiDoc™ Touch Image System (Bio-Rad)
Figure generated from this original image: Fig 15b - replicate 3

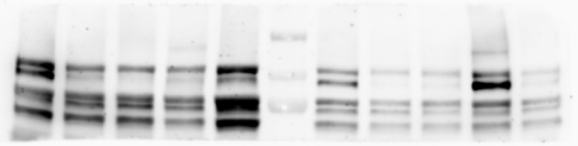

Identity of the experimental samples: total muscle lysates
Image captured by using ChemiDoc™ Touch Image System (Bio-Rad)
Figure generated from this original image: Fig 9a/b (GAPDH) - replicate 1

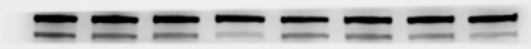

Identity of the experimental samples: total muscle lysates
Image captured by using ChemiDoc™ Touch Image System (Bio-Rad)
Figure generated from this original image: Fig 9a/b (GAPDH) - replicate 2

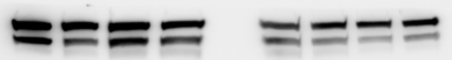

Identity of the experimental samples: total muscle lysates
Image captured by using ChemiDoc™ Touch Image System (Bio-Rad)
Figure generated from this original image: Fig 9a/b (GAPDH) - replicate 3

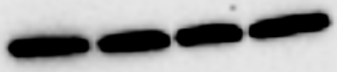

Identity of the experimental samples: total muscle lysates
Image captured by using ChemiDoc™ Touch Image System (Bio-Rad)
Figure generated from this original image: Fig 12,13,14,15 a/b - replicate 1

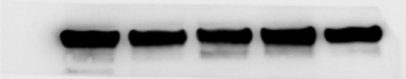

Identity of the experimental samples: total muscle lysates
Image captured by using ChemiDoc™ Touch Image System (Bio-Rad)
Figure generated from this original image: Fig 12,13,14,15 a/b - replicate 2

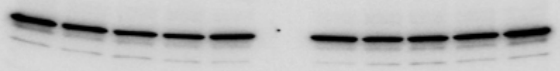

Identity of the experimental samples: total muscle lysates
Image captured by using ChemiDoc™ Touch Image System (Bio-Rad)
Figure generated from this original image: Fig 12,13,14,15 a/b - replicate 3

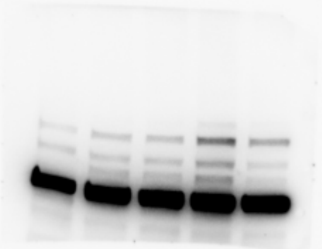

Supplement: Supplementary file 1 [file biomedicines-11-02334-s001.zip › Figure S6.pdf]
