# Supplementary material for: Metformin Protects Rat Skeletal Muscle from Physical Exercise-Induced Injury
Source: Biomedicines. 2023 Aug 22;11(9):2334. doi: 10.3390/biomedicines11092334 (PMC10525561; doi:10.3390/biomedicines11092334)
Supplement: Supplementary file 1 [file biomedicines-11-02334-s001.zip › Table S1.pdf]

Table S1: List of primary antibodies employed for Western Blot (WB) and Immunohistochemical Analysis (IHC).

**Primary Antibody**	**Source**	**Dilution /Application**	**Supplier**
Acetyl-CoA Carboxylase beta (ACCß)	rabbit	1:1000 / WB	Cell Signaling 3662
Phospho-Acetyl CoA Carboxylase (p-ACC) (Ser79)	rabbit	1:1000 / WB	Cell Signaling 3661
Protein Kinase B (AKT)	mouse	1:2000 / WB	Cell Signaling 2920
Phospho-Protein Kinase B (p-AKT) (Ser473)	rabbit	1:1000 / WB	Cell Signaling 9271
Activating Adenosine Monophosphate-activated Protein Kinase (AMPK)	rabbit	1:1000 / WB	Cell Signaling 2532
Phospho- Activating Adenosine Monophosphate-activated Protein Kinase (p-AMPK) (Thr172)	rabbit	1:1000 / WB	Cell Signaling 2535
Cytochrome C (Cyt C)	mouse	1:1000 / WB	Invitrogen 45-6100
Glyceraldehyde-3-Phosphate Dehydrogenase (GAPDH)	mouse	1:25000 / WB	Ambion AM4300
Glycogen Synthase Kinase 3 beta (GSK3ß)	rabbit	1:1000 / WB	Cell Signaling 9315
Phospho-Glycogen Synthase Kinase 3 beta (p-GSK3ß) (Ser9)	rabbit	1:1000 / WB	Cell Signaling 9323
Mammalian Target of Rapamycin (mTOR)	mouse	1:1000 / WB	Cell Signaling 4517
Phospho-Mammalian Target of Rapamycin (p-mTOR) (Ser2448)	rabbit	1:1000 / WB	Cell Signaling 2971
Myogenic Factor 5 (Myf5)	rabbit	1:10000 / WB<br>1:500 / IHC	Abcam ab125078
Myosin Heavy Chain 1/2 (MYH1/2)	mouse	1:500 / WB	Santa Cruz sc-53088
Myosin Heavy Chain 1/2/3 (MYH1/2/3)	mouse	1:50 / IHC	Santa Cruz sc-53092
Myoblast Differentiation Protein (MyoD)	mouse	1:500 / WB<br>1:100 /IHC	BD Pharmingen 554130
Myogenin	mouse	1:50 / IHC	Abcam ab1835
Paired Box 7 (PAX7)	rabbit	1:1000 / WB<br>1:200 / IHC	Abcam ab34360

**Primary Antibody**	**Source**	**Dilution /Application**	**Supplier**
Peroxisome Proliferator-Activated Receptor gamma Coactivator-1 alpha (PGC-1α)	rabbit	1:1000 /WB	Abcam ab54481
Ribosomal Protein S6 Kinase (p70S6K)	mouse	1:1000 /WB	Santa Cruz sc-8418
Phospho-Ribosomal Protein S6 Kinase (p-p70S6K) (Thr389)	mouse	1:1000 / WB	Cell Signaling 9206
vinculin	mouse	1:1000 /WB	Santa Cruz sc-25336
